# Hypersensitive Transport in Photonic Crystals with Accidental Spatial Degeneracies

**DOI:** 10.1038/srep22169

**Published:** 2016-02-23

**Authors:** Eleana Makri, Kyle Smith, Andrey Chabanov, Ilya Vitebskiy, Tsampikos Kottos

**Affiliations:** 1 Department of Physics,Wesleyan University, Middletown, CT-06459, USA; 2 Department of Physics and Astronomy, University of Texas at San Antonio, TX-78249, USA; 3 Air Force Research Laboratory, Sensors Directorate, Wright Patterson Air Force Base, OH-45433, USA

## Abstract

A localized mode in a photonic layered structure can develop nodal points (nodal planes), where the oscillating electric field is negligible. Placing a thin metallic layer at such a nodal point results in the phenomenon of induced transmission. Here we demonstrate that if the nodal point is not a point of symmetry, then even a tiny alteration of the permittivity in the vicinity of the metallic layer drastically suppresses the localized mode along with the resonant transmission. This renders the layered structure highly reflective within a broad frequency range. Applications of this hypersensitive transport for optical and microwave limiting and switching are discussed.

One of the main technological and fundamental challenges of our days is the design of electromagnetic architectures that allow for an efficient manipulation of the amplitude, phase, polarization, or direction of electromagnetic signals. Management of these features can lead to many diverse applications ranging from optical^1^ and microwave communications^2^, sensors and power limiters^3^, to energy harvesting, switching, and optical computing^4^. In this endeavor, the enhancement and control of the interaction between electromagnetic radiation and matter is of utmost importance.

An efficient way to achieve this enhancement is via localized modes supported by defect layers embedded in a layered photonic structure. Such localized modes develop nodal points where the amplitude of the oscillating electric field is very small. Placing a thin metallic nanolayer at such positions will have nearly no effect on the localized mode and the resonance transmission associated with this mode. This is the well-known phenomenon of induced transmission (see, for example^5^,^6^,^7^,^8^, and references therein). By comparison a stand-alone metallic nanolayer of the same thickness is totally opaque at the same frequency range which explains the term “induced transmission”^5^. Here we argue that a small perturbation 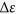 in the permittivity of a layer(s) nearby to the metallic nanolayer, can drastically affect the localized mode and resonance transmission associated with it. Depending on the nodal point symmetry, there are three possible scenarios: (a) the nodal point (with the metallic nanolayer) coincide with the mirror plane of the layered structure before and after the perturbation; (b) the nodal point coincide with the mirror plane in the original configuration, but the perturbation destroys this symmetry; and (c) the nodal point of the localized mode with the metallic nanolayer is not a symmetry point, neither before nor after the perturbation, in which case the coincidence of the metallic nanolayer and the node of the unperturbed localized mode can be viewed as *accidental spatial degeneracy* (ASD). In the case (a), the symmetric alteration of the layered structure results simply in a shift of the localized mode frequency. The metallic nanolayer still coincides with the nodal point of the localized mode at the shifted frequency and hence does not affect the resonant transmission at that frequency. In the cases (b) and (c), the nodal point of the localized mode shifts away from the metallic nanolayer, which can result in a dramatic suppression of the localized mode, along with the resonant transmission. In either case, the layered structure becomes opaque at any frequency. Due to the presence of the metallic nanolayer, the abrupt transition from resonant transmission to *broadband opacity* can be caused by just a tiny change (few percentile point) of the permittivity 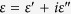 of one of the dielectric layers of the defect cavity, which justifies the use of the term *hypersensitivity*. The above feature equally applies to the cases (b) and (c), but with one important exception, when the permittivity alteration 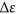 is self-induced by the localized mode. Typically, a self-induced change in the permittivity is associated with nonlinear effects, heating, etc. If the permittivity change 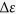 is indeed self-induced, the transition from resonant transmission to broadband opacity is very pronounced and abrupt in the case (c) of accidental spatial degeneracy as compared to the case (b), where the unperturbed layered structure is symmetric.

In most applications of metallo-dielectric layered structures (see for example^5^,^9^) the abovementioned hypersensitive transport characteristics of asymmetric configurations to a self-induced alteration of the refractive index would be undesirable and counterproductive. In this paper we take an alternative viewpoint. We demonstrate how such hypersensitive transport can be used in microwave (and optical) limiters, and we show that it can dramatically enhance their performance. As an example, we consider a microwave limiter based on an asymmetric metal-dielectric layered structure supporting a localized mode with *ASD*. We show that even a small self-induced alteration of the refractive index at the neighborhood of the maxima of the localized mode produces an abrupt transition from resonant transmission for low-level radiation to high broadband reflectivity for high-level radiation. On the other hand, if the asymmetric permittivity alteration is caused by external physical action, such as, asymmetric mechanical stress, electric field etc., rather than being self-induced by the localized mode, the abovementioned hypersensitivity will not be related to the ASD, and it will be equally strong in the setting (b) and (c). This effect can be used in switches, modulators and sensors.

It is important that the induced transmission and its hypersensitivity to the incident electromagnetic wave intensity in asymmetric metal-dielectric photonic structures are significant only in cases where the imaginary permittivity of the metallic nano-layer is large. This critical condition is satisfied at frequencies starting from microwave and up to the mid infrared.

Consider a 1D photonic crystal (PC) consisting of two lossless Bragg gratings (BG), with constitutive components different for each grating, as shown in Fig. 1. The refraction indices and thicknesses of the bilayers of the left BG (LBG) layers are 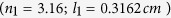 and 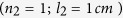 and those of the right BG (RBG) are 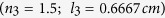; and 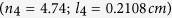 respectively. The interface between the two gratings constitutes an asymmetric cavity. The periodic modulation of the index of refraction of each grating is engineered in a way that both of them have the same band-gap structure, which is just a matter of convenience. The cavity consists of two different quarter-wave layers with 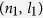 and 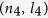 and a thin metallic nanolayer between them with thickness 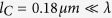 and permittivity 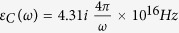. Under typical circumstances the permittivity of each of the two layers of the cavity is affected differently by an external perturbation. For example the left layer 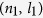 can be more sensitive to high-level radiation than the right layer. The defect cavity supports a localized mode with a frequency *f*_*r*_ located in the middle of the photonic band-gap and whose nodal point coincide with the metallic nanolayer. Evidently, this cavity is asymmetric, which corresponds to the case (c) described above.

The transmission 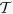, reflection 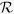, and absorption 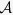 are calculated via the transfer matrix approach. The latter connects the amplitudes of forward and backward propagating waves on the left and the right domains outside of the PC. At the 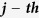 layer inside the structure, and also outside of the PC, a time-harmonic field of frequency ***ω*** satisfies the Helmholtz equation:


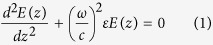


where 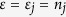
^2^ is the permittivity of the *j*-th layer (*ε* = 1 for the vacuum). At the *j*-th layer, Eq. (1) admits solutions of the form 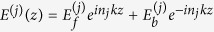, where 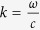 is the wavevector at the vacuum. Outside the PC, Eq. (1) admits the solution 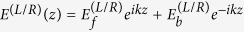. The continuity of the field and its derivative at the interface between two layers (or a layer and the vacuum) can be expressed in terms of the total transfer matrix 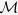 which connects the forward and backward amplitudes on the left (L) and right (R) of the PC:


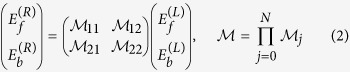


where *N* is the total number of layers. The single-layer transfer matrix 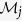 connects the field amplitudes of the *j*-th and the 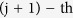 layers i.e. 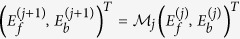. Thus the transfer matrix approach allows us also to construct the field 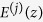 at each layer, provided that appropriate scattering boundary conditions are imposed. The latter, for a left incident wave, take the form 
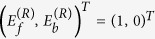
. It is easy to show that 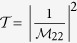, 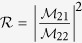, and 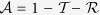
10,11.

We start our analysis with the investigation of the transmission spectra of each of the two mirrors. Their dispersion relation 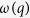 is calculated using the transfer matrix of one bilayer 
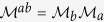
11


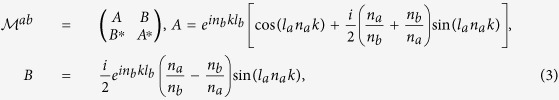


where the indices a and b indicate the layers 1 and 2 (3 and 4) associated with the LBG (RBG).

Propagating waves in each grating correspond to frequencies 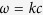 for which^11^


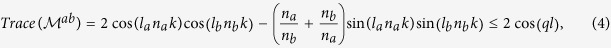


where the total width of the bilayer 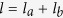 defines the periodicity of the LBG (for 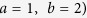 or the RBG (for 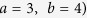. Direct inspection of Eq. (4) indicates that the dispersion relations 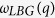 and 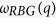 are identical as long as 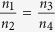 and 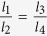. Once turned to finite photonic structures, both LBG and RBG will share the same band-gap structure of the transmission spectrum, as long as these conditions are satisfied. Below we consider that each BG consists of five (quarter-wavelength) bilayers.

In Fig. 1b we show the transmission spectrum 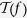 of our PC for 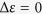. The position of the band-edges is nicely described by Eq. (4). Moreover a resonant mode with 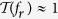 at resonance frequency 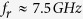, in the middle of the band-gap, has been created. The resonant mode is localized at the vicinity of the defect cavity and decays exponentially inside the two mirrors due to destructive interferences from the layers (blue profile at Fig. 1). The electric field 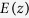 has a nodal point at the position of the metallic layer (blue profile at Fig. 1a). Thus the resonant localized mode is unaffected by the presence of the lossy layer and the entire PC is completely transparent at 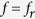 (see Fig. 1b). Furthermore, the lack of mirror symmetry ensures that the ASD occurs only for the resonance mode *f*_*r*_. For all other (Fabry-Perot) resonances with frequencies 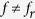 the electric field distribution has finite amplitude at the position of the metallic layer leading to large reflection 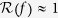 (see discussion below) and vanishing transmission 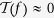.

Moreover, any small perturbation (say, due to heating), which will change the permittivity of any of the two layers of the defect cavity (say the left one) by 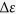, will engage immediately the metallic nanolayer and lift the ASD of the resonance localized mode. In other words, the electric field will no longer have a nodal point at the position of the metallic layer (see red profile at Fig. 1a). This will trigger various competing mechanisms. On the one hand, it will increase the impendence mismatch and thus it will enhance the reflection. This mechanism is present whenever the electric field interacts with the metallic layer, even for 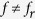. On the other hand, it will lead to an increase of absorption. One can estimate the effect of these two competing mechanisms by analyzing the transport from a single lossy *δ*-like defect with permittivity 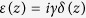. In this case, we have that:


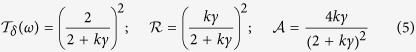


which indicates that a lossy defect is a source of increased absorption but at the same time a way to enhance reflection. Also note that the absorption is not a monotonic function of the tangent loss parameter *γ*. Rather it takes its maximum value 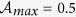 at 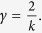


The second mechanism applies only for resonant transport. In this case, the bulk losses due to the strong interaction of the electric field with the metallic layer compete with the losses due to the leakage from the boundaries of the structure. The former are proportional to the field intensity at the position of the lossy defect, while the latter depend on the coupling of the resonant mode to the free space via the boundary of the PC. As 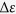 increases, the bulk losses overrun the losses due to the boundary leakage and eventually spoil the resonance (see red electric field in Fig. 1). Thus, photons do not dwell in the resonant mode and therefore cannot be absorbed by the metallic layer i.e. the absorption 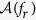 diminishes while 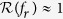, and 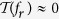.

In Fig. 2 we report the transmission 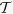, reflection 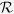, and absorption 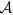 of our PC, versus frequency and versus the relative permittivity change 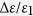 occurring at a single (left of the metallic nanolayer) layer. For small 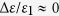, 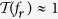 (Fig. 2a) while 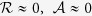 respectively (Fig. 2b,c). As 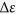 increases, the absorption is initially increasing (see peak at 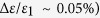 but for 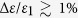 it starts decreasing reaching values as low as −40 dB. For even larger 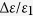, the structure becomes completely reflective (see Fig. 2b).

The hypersensitivity of the transport characteristics of our composite structure to small permittivity changes 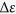 can find various applications including sensors, switches, power modulators, etc. Here, however, we will discuss the advantages to implement our PC as an efficient energy limiter. These are devices that protect electromagnetic sensors from high-energy radiation, while at the same time they are transparent to low energy radiation^12^,^13^,^14^,^15^,^16^,^17^,^18^,^19^,^20^,^21^,^22^,^23^,^24^. Typically this protection is achieved via the absorption of the incident energy from the limiter, which turns opaque. At the same time this excessive energy overheats the limiter and leads to its self-destruction. Recently, however, the concept of reflective limiters has been introduced^25^,^26^. These structures consist of a BG with one lossy defect layer, which undergoes a uniform self-induced permittivity change. The proposal for limiting action was based on the phenomenon of resonant transmission via a localized (defect) mode. The defect mode is transmissive at low-energy incident pulses, while it becomes highly reflective (and not absorbing) at high-energy pulses. Nevertheless, this proposal suffers from one drawback; the limiting action requires several orders of magnitude change of the permittivity of the lossy defect. Instead, our design requires changes of only a few percentage points in the permittivity of one composite layer in order to provide limiting action. Importantly, the high reflectivity for the high intensity input persists within a broad frequency range – not just within a photonic band-gap is was the case in refs 25,26.

We consider the PC of Fig. 1. We further assume, for the sake of the discussion, that the left layer of the defect cavity has a permittivity which depends on temperature (*T*) variations as 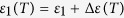 where for simplicity we consider that 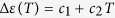. Since the layer on the right is composed of a different material, in general, we expect a different variation of its permittivity with the temperature. For simplicity, we assume that the right layer is much more resilient to the changes in temperature and thus we will keep its permittivity constant 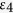. There are various physical mechanisms that can lead to the heating of the dielectric layer. For example, it can originate from the heating of the nearby metallic nanolayer or from the presence of a small 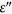 at the permittivity of the dielectric layer itself (which is usually the case in practical situations) or from a combination of these two physical mechanisms.

The rate equation that determines the temporal behavior of the temperature 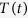 at the cavity is^26^:


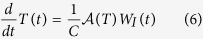


where 
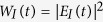
 is the incident pulse intensity, which is a given function of time, *C* is the heat capacity, and 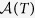 is the temperature dependent absorption coefficient of the asymmetric cavity. A numerical integration of Eq. (6) (for a given pulse profile 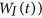 allows us to evaluate the temperature 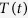 and from there the permittivity variations 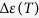 which are reported in Fig. 3a as a percentage change 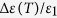. Then 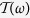, 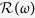 and 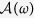 are calculated using transfer matrices as a function of pulse duration *t* (see Fig. 3b–d). We find that 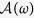 (Fig. 3b) initially increases and reaches some maximum value around 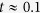 corresponding to very small permittivity changes 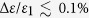. Further increase of 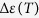 leads to an abrupt decay of 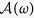 for resonance frequencies to values smaller than −30 dB. The off-resonance values already have absorption that is below −60 dBs. At the same time the transmission (Fig. 3c) decays while the reflection (Fig. 3d) reaches unity. Therefore our photonic structure acts as a hypersensitive reflective microwave limiter- it will turn highly reflective within a broad frequency range for very small relative permittivity changes ~0.5%. This behavior has to be contrasted with the proposal of ref. 25 where a limiting action is triggered only when the variation (due to heating) of the refraction index 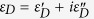
of a defect lossy layer, which is embedded in a Bragg grating, is many orders of magnitude. The outcome of these calculations is also reported in the inset of Fig. 3a by referring to 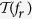, 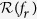 and 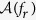 at resonance frequency versus the relative change of the permittivity. For these simulations we have used a BG with the same constitutive layers as the LBG of our PC. The lossy defect layer is placed in the middle of the grating and has 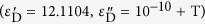. We see that the reflective limiting action occurs when the permittivity changes of the lossy defect layer are more than seven orders of magnitude.

In conclusion, we have introduced a photonic layered structure design with hypersensitive transport characteristics. This layered structure consists of an asymmetric dielectric cavity incorporating a metallic nanolayer and sandwiched between two Bragg mirrors. When the metallic nano-layer coincides with the nodal point of the localized mode, the system develops the phenomenon of induced transmission. However, even a small change in 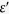 and/or 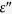 in one of the dielectric layers of the asymmetric cavity abruptly suppresses the localized mode and renders the layered structure highly reflective at all frequencies – not just at frequencies of the photonic band gap. Furthermore, we have shown that these metal-dielectric structures can be used as hypersensitive microwave (or optical) limiters. Specifically, at low incident wave intensity, these structures support a narrow-band transmission. If the electromagnetic wave intensity and/or fluence exceed certain level, even a small self-induced change (due to non-linearities or heating effects) in the refractive index of the asymmetric layer causes an abrupt transition to a broadband reflectivity. The proposed design can be adjusted to different frequency ranges starting from microwave frequencies and up to the mid infrared. The main physical requirement is that the imaginary part 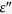 of the metallic nanolayer is large. The concept of hypersensitive layered structures can be applied not only to electromagnetic waves but also to acoustic waves, matter-waves etc.

## Additional Information

**How to cite this article**: Makri, E. *et al.* Hypersensitive Transport in Photonic Crystals with Accidental Spatial Degeneracies. *Sci. Rep.* **6**, 22169; doi: 10.1038/srep22169 (2016).

## Figures and Tables

**Figure 1:**
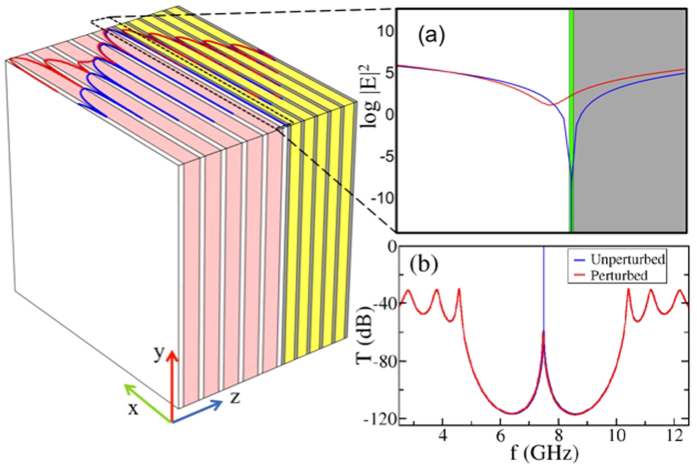
Metal-dielectric PC consisting of two BG with different constitutive bi-layers (white and orange for the LBG and grey and yellow for the RBG). The blue and the red curves in the upper facet of the structure show the resonant field profile for the unperturbed and perturbed structures, respectively. In the perturbed structure, a small 2% increase of the permittivity of the white layer completely suppressed the resonant mode (red curve in the upper facet of the PC—main figure). The dashed box indicates the position of the asymmetric defect cavity at the interface of the two mirrors. (**a**) A magnified view of the asymmetric cavity. The cavity consists of two dielectric layers (white and gray) separated by a metallic nanolayer (green) in the middle. A magnification of the field distribution around it (blue and red curves corresponding to the unperturbed and perturbed structures) is also reported. Notice the shift of the nodal point in the red curve, which results in engaging the metallic nanolayer and lead to a suppression of the resonant transmission, as shown in (**b**). (**b**) Transmission dispersion of the unperturbed structure (blue curve) and perturbed structure (red curve). The field profile at the frequency of resonant transmission for either case is shown in (**a**). Note that in the case of symmetric metal-dielectric cavity, the perturbation would lead to a simple shift of the resonant transmission, not to its suppression.

**Figure 2:**
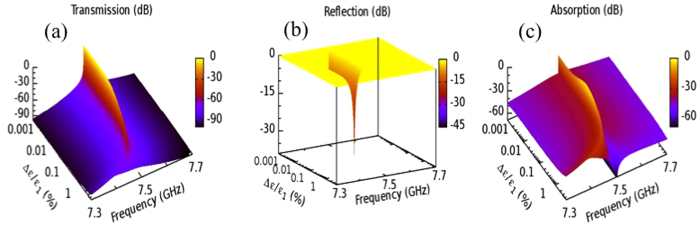
(**a**) Transmission versus frequency and change in permittivity in the vicinity of the resonant transmission. For 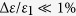, the resonant transmission 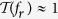. As 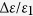 grows, the transmission drops below −40 dB. (**b**) The reflection 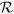 versus frequency and 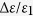. Low values of the resonant reflectivity 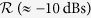 only occur for small 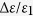. For 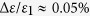 the reflection becomes almost zero 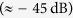 while the absorption gets its maximum value (see subfigure (**c**)). For larger 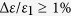, our structure becomes highly reflective. (**c**) The absorption versus frequency and 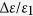. For 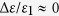 the absorption 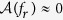 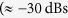, see color coding). For small 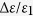 it increases and reaches a maximum at 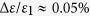. As 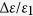 increases further, 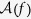 decays abruptly and takes values smaller than –40 dBs.

**Figure 3:**
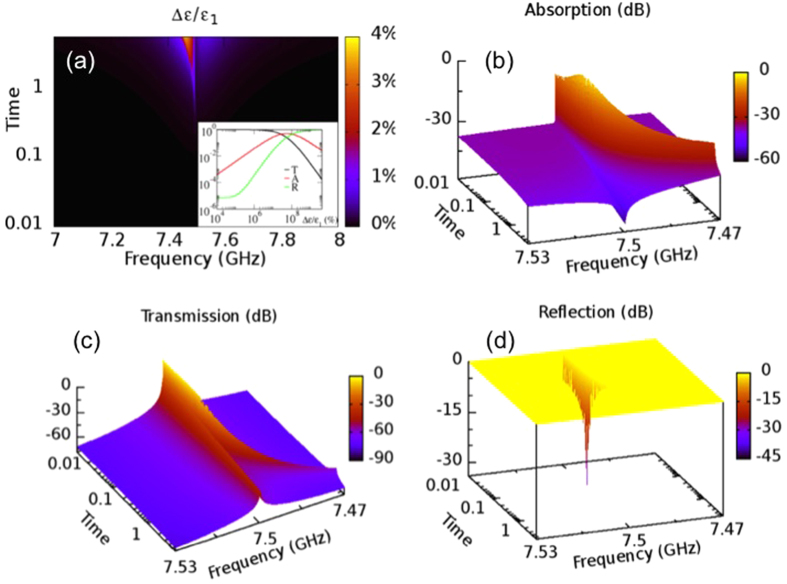
(**a**) A density plot of the change of permittivity 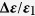 of the left layer of the defect cavity in the vicinity of transmission resonance as a function of the pulse duration. Inset: the transmission T, reflection R and absorption A at resonance frequency versus 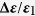 for a reflective limiter of refs 25,26. (**b**) The absorption; (**c**) the transmission; and (**d**) the reflection versus pulse duration, for a frequency window around the resonance mode.
